# Epigallocatechin Gallate-Modified Gelatin Sponges Treated by Vacuum Heating as a Novel Scaffold for Bone Tissue Engineering

**DOI:** 10.3390/molecules23040876

**Published:** 2018-04-11

**Authors:** Yoshitomo Honda, Yoshihiro Takeda, Peiqi Li, Anqi Huang, Satoshi Sasayama, Eiki Hara, Naoya Uemura, Mamoru Ueda, Masanori Hashimoto, Kenji Arita, Naoyuki Matsumoto, Yoshiya Hashimoto, Shunsuke Baba, Tomonari Tanaka

**Affiliations:** 1 Institute of Dental Research, Osaka Dental University, 8-1, Kuzuhahanazonocho, Hirakata, Osaka 573-1121, Japan; 2 Department of Oral Implantology, Osaka Dental University, 1-5-17, Otemae, Chuo-ku, Osaka 540-0008, Japan; takeda-y@cc.osaka-dent.ac.jp (Y.T.); lipeiqi999@gmail.com (P.L.); huanganqident@gmail.com (A.H.); satoshi_sasayama@mac.com (S.S.); gateway8020@yahoo.co.jp (N.U.); baba-s@cc.osaka-dent.ac.jp (S.B.); 3 Department of Orthodontics, Osaka Dental University, 1-5-17, Otemae, Chuo-ku, Osaka 540-0008, Japan; eikihara@icloud.com (E.H.); naoyuki@cc.osaka-dent.ac.jp (N.M.); 4 First Department of Oral and Maxillofacial Surgery, Osaka Dental University, 1-5-17, Otemae, Chuo-ku, Osaka 540-0008, Japan; huzan19890823@yahoo.co.jp; 5 Department of Oral Health Sciences, Faculty of Health Sciences, Osaka Dental University; 1-4-4, Makinohonmachi, Hirakata, Osaka 573-1144, Japan; hashimoto@cc.osaka-dent.ac.jp; 6 Department of Pediatric Dentistry, Osaka Dental University, 1-5-17, Otemae, Chuo-ku, Osaka 540-0008, Japan; arita-k@cc.osaka-dent.ac.jp; 7 Department of Biomaterials, Osaka Dental University, 8-1, Kuzuhahanazonocho, Hirakata, Osaka 573-1121, Japan; yoshiya@cc.osaka-dent.ac.jp; 8 Graduate School of Science and Technology, Kyoto Institute of Technology, Matsugasaki, Sakyo-ku, Kyoto 606-8585, Japan

**Keywords:** catechin, EGCG, vacuum heating, dehydrothermal, UMR106, gelatin, scaffold, bone formation, rat calvaria

## Abstract

Chemical modification of gelatin using epigallocatechin gallate (EGCG) promotes bone formation in vivo. However, further improvements are required to increase the mechanical strength and bone-forming ability of fabricated EGCG-modified gelatin sponges (EGCG-GS) for practical applications in regenerative therapy. In the present study, we investigated whether vacuum heating-induced dehydrothermal cross-linking of EGCG-GS enhances bone formation in critical-sized rat calvarial defects. The bone-forming ability of vacuum-heated EGCG-GS (vhEGCG-GS) and other sponges was evaluated by micro-computed tomography and histological staining. The degradation of sponges was assessed using protein assays, and cell morphology and proliferation were verified by scanning electron microscopy and immunostaining using osteoblastic UMR106 cells in vitro. Four weeks after the implantation of sponges, greater bone formation was detected for vhEGCG-GS than for EGCG-GS or vacuum-heated gelatin sponges (dehydrothermal cross-linked sponges without EGCG). In vitro experiments revealed that the relatively low degradability of vhEGCG-GS supports cell attachment, proliferation, and cell–cell communication on the matrix. These findings suggest that vacuum heating enhanced the bone forming ability of EGCG-GS, possibly via the dehydrothermal cross-linking of EGCG-GS, which provides a scaffold for cells, and by maintaining the pharmacological effect of EGCG.

## 1. Introduction

Cost is a major limitation for further progress in the field of regenerative therapy [1]. The functionalization of cost-effective and abundant molecules is an attractive strategy to produce useful materials to overcome this issue. Tea from the leaf of *Camellia sinensis* is widely consumed on a daily basis [2]. Green and oolong teas contain a variety of polyphenol catechins, such as epicatechin, epigallocatechin gallate (EGCG), and gallocatechin gallate (GCG) [3,4]. EGCG, the most abundant catechin in green tea, has greater pharmacological effects than those of other catechins [5]. EGCG exhibits various biological activities, including anti-bacterial [6], anti-carcinogenic [7], anti-oxidative stress [8], and anti-inflammatory [9] activities. Additionally, the safety [2] and cost-effectiveness [10] of EGCG support its broad medical usage. Therefore, EGCG is already recognized as a prospective health-promoting agent in various medical fields, with applications in diabetes [11], Alzheimer’s disease [12], obesity [13], cardiovascular disease [14], cancer [3,15], bone disease [16], and oral disease [17].

Bone mass is mainly controlled by the balance between bone formation by osteoblasts and bone resorption by osteoclasts. There is increasing evidence that EGCG effectively induces osteoblast differentiation in multipotent progenitor cells, such as human mesenchymal stem cells [18], mouse mesenchymal stem cells [19], and human dedifferentiated fat cells [20]. EGCG enhances mineralization in human osteoblast-like cells [5,21]. However, an adequate strategy to achieve pharmacological effects of EGCG in bone regeneration therapy in vivo is still controversial. Several techniques have been under intensive investigation to optimize conjugation, cross-linking, and matrix incorporation [22,23,24,25]. Recently, we have reported that the concomitant use of non-cross-linked EGCG and a dehydrothermal (DHT) cross-linked gelatin sponge had a negligible effect on the induction of bone formation in critical-sized mouse calvaria [23]. In contrast, a gelatin sponge chemically modified with EGCG induced superior bone formation [23]. However, the fabricated EGCG-modified gelatin sponge (EGCG-GS) lacked sufficient mechanical strength to sustain the scaffold properties. Further improvements are imperative to enhance its bone-forming ability compared with that of autogenous bone grafting, a gold standard technique in bone regenerative therapy [1,26]. 

Gelatin, a random coiled protein denatured from collagen [27], is widely used in the food industry [19] and biomedical field owing to its low cost, biocompatibility, biodegradability, and low immunogenicity [28]. Gelatin exhibits cellular attachment [29]; its susceptibility to enzymatic degradation can be used to generate controlled release materials [30]. Despite these beneficial properties for regenerative therapy, gelatin sponges generally show poor mechanical strength and low enzyme resistance due to the disruption of natural cross-linking and assembly structure of collagen [31]. To overcome these disadvantages, gelatin sponges are frequently strengthened by cross-linking techniques, including chemical [32], enzymatic [33], and physical methods [24]. Among physical cross-linking methods, DHT cross-linking induced by vacuum heating strengthens the mechanical properties of gelatin hydrogels [34]. Although vacuum heating is a potential technique to stabilize the gelatin matrix in EGCG-GS, EGCG is heat-sensitive and produces epimers [4,35], which may counteract its pharmacological effect. To the best of our knowledge, attempts to apply vacuum heating to biomaterials containing EGCG are limited; it is not clear whether this procedure retains the pharmacological effect of EGCG and augments the bone-forming ability of EGCG-GS. 

In the present study, we compared the bone-forming ability of EGCG-GS and vacuum-heated EGCG-GSs (vhEGCG-GSs) using 9-mm critical-sized defects of rat calvaria and subsequent micro-computed tomographic and histological analyses. To elucidate the mechanisms underlying bone formation via vhEGCG-GS, rat osteoblastic UMR106 cells were cultured on the sponge in vitro. Cell morphology, proliferation, and cell–cell communication were assessed by scanning electron microscopy (SEM) and immunostaining. 

## 2. Results

### 2.1. Characterization of Sponges

**Figure 1:**
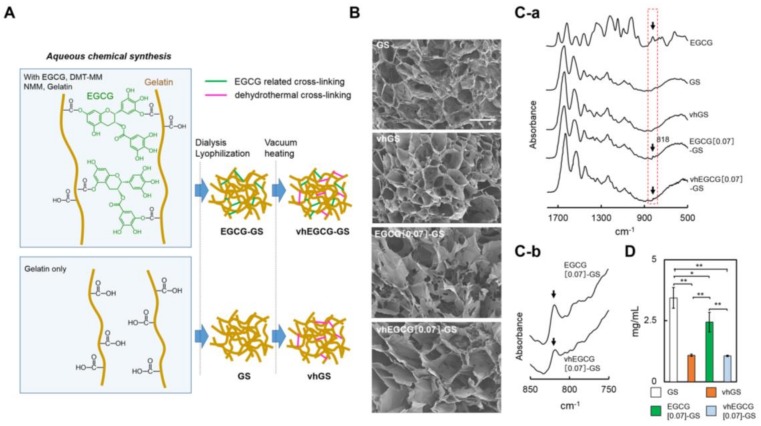
Schematic diagram and characterization of sponges. (**A**) Schematic diagram of sponge preparation. (**B**) Scanning electron microscope (SEM) images of the sponges. Bar: 250 µm. (**C-a**,**C-b**): Low and high magnification of Fourier-transform infrared spectra. Arrows in (**C-a**,**C-b**): specific spectra, 818 cm^−1^, of epigallocatechin-gallate (EGCG). (**D**) Degradation of the sponges in distilled water after 90 h of incubation at 37 °C. ** *p* < 0.01 and * *p* < 0.05, mean and standard deviation (SD) (*n* = 5, ANOVA with Tukey–Kramer test). DMT-MM: 4-(4,6-dimethoxy-1,3,5-triazin-2-yl)-4-methyl-morpholinium chloride. NMM: *N*-methylmorpholine.

Figure 1A shows a schematic diagram of the synthesis process for EGCG-GS, vhEGCG-GSs, gelatin sponge (GS), and vhGS. The name and synthesis conditions for each sample are briefly listed in Table 1. Hereafter, N in EGCG[N]-GS and vhEGCG[N]-GS represents the dose of EGCG at synthesis. To highlight the comparison between EGCG[0.07]-GS and vhEGCG[0.07]-GS, their characteristics have been described in the main text, whereas those of vhEGCG[0.7]-GS and vhEGCG[6.7]-GS are included in the Supplementary Materials. As with GS and vhGS, both EGCG[0.07]-GS and vhEGCG[0.07]-GS exhibited a spongy morphology with a highly porous structure (Figure 1B). The specific Fourier-transform infrared spectra of EGCG (818 cm^−1^; C–H bending of aromatic ring) [36] were detected for both EGCG[0.07]-GS and vhEGCG[0.07]-GS (arrows in Figure 1C-a,C-b). We did not observe remarkable changes in the spectra after treatment by vacuum heating for EGCG-GS and GS. As reported previously [23], chemical modification of gelatin using EGCG clearly prevented the degradation of GS (EGCG-GS vs. GS in Figure 1D). Vacuum heating significantly attenuated the degradation of EGCG[0.07]-GS, yet its degradability was similar to that of vhGS. 

### 2.2. Micro-Computed Tomography and a Quantitative Analysis

**Figure 2:**
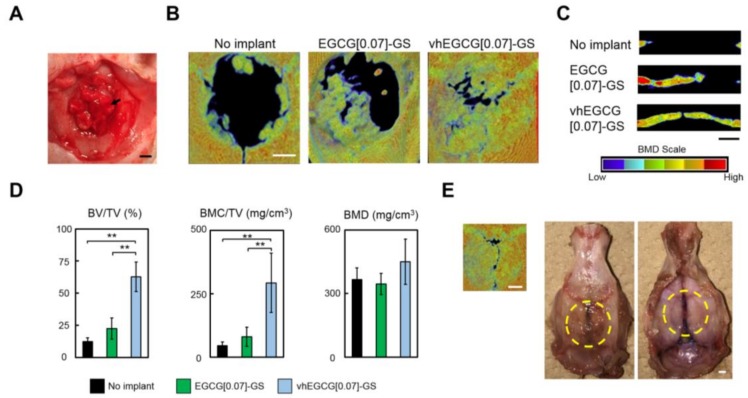
Micro-computed tomography analysis and macroscopic images of defects. (**A**) Representative macroscopic image of defects treated with sponges after implantation surgery (sponges: vhEGCG[0.07]-GS). Arrow: implanted sponge. (**B**,**C**): Vertical and lateral bone mineral density (BMD) image reconstructed by micro-computed tomography of the defects treated with or without the sponges after 4 wk of implantation. (**D**) Quantitative analysis of micro-computed tomography data for each defect: BV, bone volume; TV, total volume; BMC, bone mineral content; BMD, bone mineral density. ** *p* < 0.01, mean and SD (*n* = 5, ANOVA with Tukey-Kramer test). (**E**) Optimal sample of defect treated with vhEGCG[0.07]-GS. Vertical BMD image and macroscopic images of the calvaria. Yellow circles highlight the defect. Bars: 2 mm.

To evaluate the bone-forming ability of each material, the seven sponge pieces were implanted into calvarial defects (Figure 2A). Bone mineral density (BMD) images reconstructed by micro-computed tomography observed from vertical and lateral views revealed that radiopacity was greater for defects treated with vhEGCG[0.07]-GS than for those treated with EGCG[0.07]-GS or no implant after 4 wk (Figure 2B,C). Consistent with this qualitative analysis, although there were no significant differences in BMD in defects between vhEGCG[0.07]-GS and EGCG[0.07]-GS, bone volumes (BV) and bone mineral contents (BMC) per total volume (TV) for defects treated with vhEGCG[0.07]-GS were significantly greater than those for defects treated with EGCG[0.07]-GS. It is noteworthy that the optimal sample using vhEGCG[0.07]-GS largely closed the 9-mm (large critical size) defect within 4 wk, without any other added active agents (Figure 2E). 

**Figure 3:**
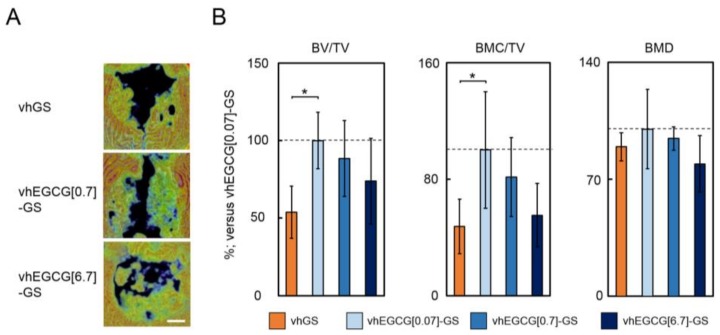
Effect of EGCG dose on the bone-forming ability of vhEGCG-GSs. (**A**) Vertical BMD image taken at 4 wk of defects treated with vhGS and vhEGCG-GSs. vhEGCG[N]-GS: N = dose of EGCG at synthesis. vhGS: vacuum-heated gelatin sponge without EGCG. Bar: 2 mm. (**B**) Quantitative analysis of micro-computed tomography data for each defect. BV, bone volume; TV, total volume; BMC, bone mineral content; BMD, bone mineral density. Broken lines: 100% lines representing the mean value for vhEGCG[0.07]-GS. * *p* < 0.05, mean and SD (*n* = 5, ANOVA with Tukey–Kramer test).

To determine whether the prominent bone formation by vhEGCG[0.07]-GS was induced by the scaffold with DHT cross-linking of gelatin or the pharmacological effect of EGCG, we altered the dose of EGCG in vhEGCG-GSs (Figure 3). The radiopacity of the defects treated with vhEGCG[0.07]-GS was significantly greater than that of the defects treated with vhGS. Although we did not detect a statistically significant difference among the vhEGCG[0.07 to 6.7]-GSs, the mean radiopacity values decreased in a dose-dependent manner (Figure 3).

### 2.3. Histological Analysis of Defects Treated with Vacuum-Heated and Non-Vacuum-Heated EGCG-Modified Gelatin Sponges

**Figure 4:**
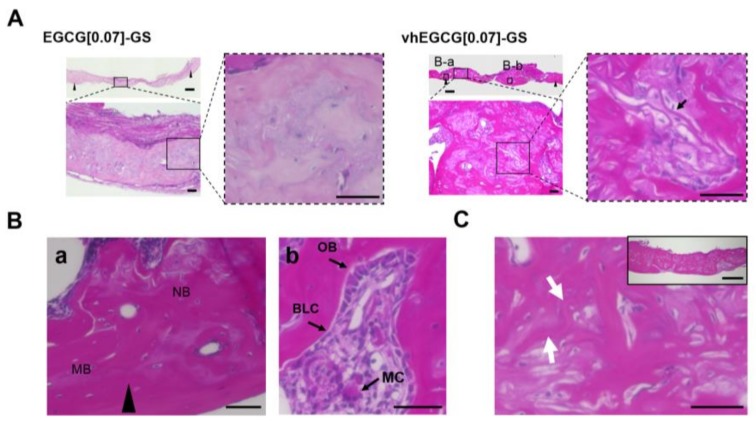
Histological images of the defect treated with sponges. (**A**) Low- and high-magnification images of defects treated with EGCG[0.07]-GS and vhEGCG[0.07]-GS, taken 4 wk after implantation. Gap between black triangles: created bone defects. (**B-a**,**B-b**): featured areas for 4B. (**B**) featured areas in 4A. MB: mother bone. NB: newly formed bone. OB: osteoblast. BLC: bone lining cell. MC: multinuclear cell. (**C**) Low and high magnification histological images of defects treated with vhEGCG[0.07]-GS, taken 8 wk after implantation. White arrows: integrated fibrous matrix of vhEGCG[0.07]-GS. Bars: 600 µm for lowest magnification images of (**A**,**C**); 50 µm for other images.

Figure 4A,B shows histological images of the defects treated with EGCG[0.07]-GS and vhEGCG[0.07]-GS after 4 wk of implantation. Consistent with the difference in degradability between materials (Figure 1D), the specific structure of EGCG[0.07]-GS was unrecognizable in the bone defect; EGCG[0.07]-GS appeared to be integrated in the newly formed bone. In contrast, a specific fibrous structure was detected in the defect treated with vhEGCG[0.07]-GS (arrow in high-magnification view of vhEGCG[0.07]-GS of Figure 4A). Numerous cells were attached to the fibrous matrices. These fibrous matrices were more highly integrated in the newly formed bone at 8 wk than at 4 wk (Figure 4C). At the margin of the defects, osteoconduction occurred from the mother bone (Figure 4Ba). Moreover, both activated osteoblast-like cells (cubical shape) and multinuclear cells (osteoclast-like cells) were found in the newly formed bone in the bone defects treated with vhEGCG[0.07]-GS (Figure 4Bb), suggesting that bone remodeling was sustained at 4 wk. 

### 2.4. Cell Attachment, Proliferation, and Communication on the Vacuum-Heated EGCG-Modified Gelatin Sponge

**Figure 5:**
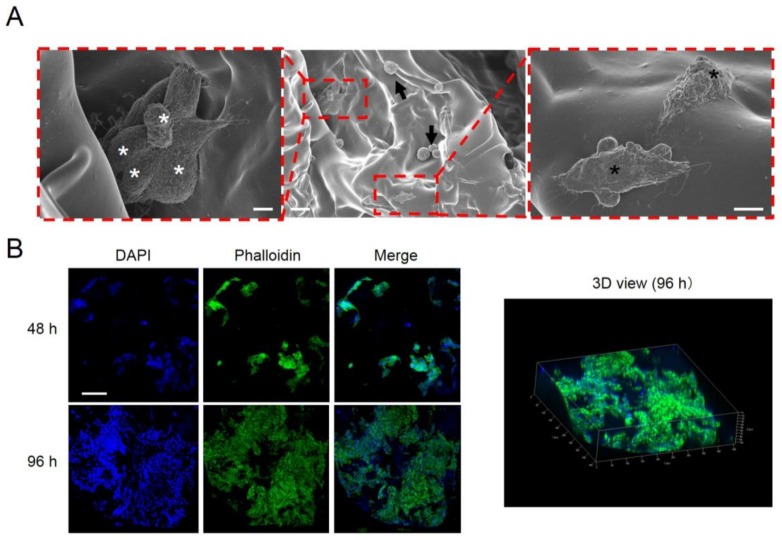
Cell behavior on the vhEGCG-GS. (**A**) Low- and high-magnification SEM images of UMR106 cells on vhEGCG[0.07]-GS after 72 h of incubation. White asterisks: aggregated cells. Black arrows: round cells. Black asterisks: spreading cells. Bars: 20 µm for low-magnification image; 5 µm for high-magnification images. (**B**) immunofluorescent staining of UMR106 cells. Bar = 100 µm.

**Figure 6:**
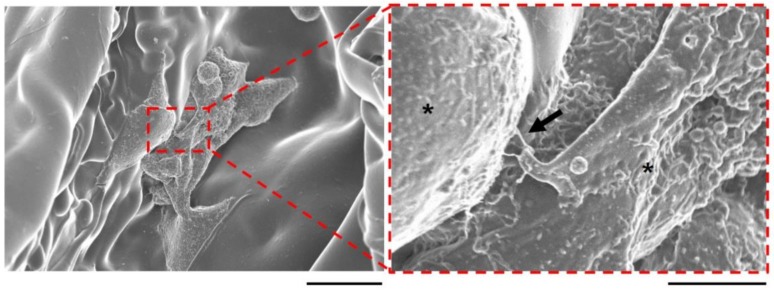
Cell–cell communication of UMR106 cells on the vhEGCG-GS. Low- and high-magnification SEM images of UMR106 cells on the vhEGCG[0.07]-GS after 72 h of incubation. Asterisks: cells. Arrow: filopodia-like structure. Bars: 25 µm for low magnification image; 5 µm for high-magnification image.

The migration of osteoblasts is associated with osteoconduction [37]. To explore the mechanism underlying the marked bone formation for vhEGCG[0.07]-GS compared with that observed for EGCG[0.07]-GS, we evaluated cell morphology and proliferation on both materials in vitro using the osteoblast-like cell line UMR106. EGCG[0.07]-GS was used as a control. However, EGCG[0.07]-GS rapidly disintegrated within 1 h in cell culture medium without stirring, resulting in difficulty of cell culture. Meanwhile, vhEGCG[0.07]-GS retained its form in the culture medium (arrows in Figure S2). After 72 h of cell culturing, we found round-shaped UMR106 cells, but also elliptic spreading cells, suggesting that the cells effectively attached to the vhEGCG[0.07]-GS (Figure 5A). We confirmed cell–cell communication with cell condensation and with the filopodia-like structure between the cells observed on the vhEGCG[0.07]-GS (left side of Figure 5A and arrow in Figure 6). Immunostaining of UMR106 cells revealed that the number of cells increased remarkably on and in the vhEGCG[0.07]-GS over time (Figure 5B). These results suggest that the vhEGCG-GS might facilitate the migration and cell–cell communication of osteoblasts from calvarial mother bone effectively, which is likely to be partially associated with the superior bone formation of vhEGCG[0.07]-GS.

## 3. Discussion

Our results demonstrate that vhEGCG[0.07]-GS exhibited greater bone-forming ability than EGCG[0.07]-GS and vhGS, indicating that the pharmacological effect of EGCG was retained, even after vacuum heating. In vitro results revealed that vacuum heating attenuated the degradation of EGCG[0.07]-GS. vhEGCG[0.07]-GS provided a sufficient scaffold for UMR106 cell attachment and proliferation as well as cell–cell communication. 

Matrix degradability is influenced by the degree of cross-linking [38]. Previous studies have reported that the vacuum heating technique adds DHT cross-linking to gelatin sponges [39] and collagen sponges [40], attenuating biodegradability. In the present study, the degradability of vhEGCG[0.07]-GS was significantly lower than that of EGCG[0.07]-GS in distilled water (Figure 1D). The EGCG[0.07]-GS readily degraded in cell culture medium within 1 h, whereas the vhEGCG[0.07]-GS retained its morphology (Figure S2). Although we were not able to verify the detailed location of DHT cross-linking in the vhEGCG-GS, these results support that the vacuum heating process effectively induces DHT cross-linking under the existence of EGCG in the sponges. 

Despite numerous studies of the pharmacological effect of EGCG, little is known about its robustness after the vacuum heating process. EGCG induces osteogenesis in multipotent progenitor cells up to a certain concentration in vitro, but this effect is gradually attenuated in a concentration-dependent manner [18,20]. Consistent with these reports, our results indicated that bone formation was greater for vhEGCG[0.07]-GS than for vhGS (Figure 3). However, the mean BV/TV and BMC/TV appeared to decrease as the EGCG dose increased, although the differences were not statistically significant. Given that the degradability of vhEGCG[0.07]-GS was similar to that of vhGS (Figure 1D), the augmentation of bone formation by vhEGCG[0.07]-GS is explained not only by its provision of a scaffold for cells, but also by its ability to retain the pharmacological effect of EGCG, even after vacuum heating. 

We previously reported that the DHT treatment of the octacalcium phosphate/collagen sponge increased cross-linking and augmented its bone-forming ability [41]. In contrast, Wada et al. showed that more highly cross-linked gelatin/calcium phosphate showed less bone formation than a less cross-linked form [42]. Low degradability of biomaterials occasionally results in the presence of residual material, which may cause contamination in the bone replacement therapy [43]. Based on these results, it is likely that the control of vacuum heating conditions alters the degree of cross-linking and degradability, affecting the results of bone replacement therapy. In the present study, as a first step, only a single vacuum heating condition (150 °C, 24 h) was used to prepare vhEGCG-GSs; this condition was determined based on our previous study of the preparation of octacalcium phosphate/gelatin sponges [44]. Although we obtained effective bone formation without a loss of the pharmacological effect of EGCG, thereby providing a scaffold for cells in these vacuum heating conditions, further optimization of the conditions might improve bone replacement therapy using vhEGCG-GS.

Most surprisingly, our vhEGCG[0.07]-GS sample effectively closed the large-sized defect (9 mm) of the rat calvaria within 4 wk based on a vertical view of the BMD, although the newly formed bone was still thinner than the original calvaria (Figure 2C,E). In our previous studies using the same rat model, the BV/TV value of bone defects treated with autogenous bone grafts at 4 wk was approximately 37.5% [45], which is obviously lower than that for vhEGCG[0.07]-GS (62.8%) (Figure 2D). The BV/TV of bone defects treated with α-tricalcium phosphate/collagen sponge was 44.7%, even at 6 wk [46]. These findings suggest that vhEGCG-GSs are promising candidates for bone regenerative medicine. However, further careful analyses of this material are imperative to verify its potential application, including analyses of long-term implantation to track the fate of the material, the detailed mechanism of bone formation, and safety. Furthermore, basic data regarding the ratio and the cross-linking properties of EGCG and gelatin will also provide beneficial information for fabricating new materials with EGCG. 

## 4. Materials and Methods 

### 4.1. Preparation of Sponges

EGCG was purchased from Bio Verde Inc. (Kyoto, Japan). 4-(4,6-Dimethoxy-1,3,5-triazin-2-yl)-4-methyl-morpholinium chloride (DMT-MM) and *N*-methylmorpholine (NMM) were purchased from Tokyo Chemical Industry Co., Ltd. (Tokyo, Japan) and Nacalai Tesque Inc. (Kyoto, Japan), respectively. Gelatin extracted from porcine skin in acidic conditions (Type A gelatin) was purchased from Sigma-Aldrich (St. Louis, MO, USA). EGCG-GS was prepared by an aqueous synthesis method reported previously [23]. In brief, gelatin (100 mg) was dissolved in warm Milli-Q water (5 mL) at 50 °C. A solution with NMM (27.5 µL), EGCG (0.07, 0.7 or 6.7 mg), and DMT-MM (69.2 mg) was stirred for 24 h at room temperature in the dark. The products were purified by dialysis (Spectra/Por7 MWCO 1000; Spectrum Labs, Rancho Dominguez, CA, USA) in Milli-Q water in the dark. The same conditions but without EGCG, DMT-MM, and NMM were used to prepare the gelation solution. After dialysis, the resulting solution was diluted to 10 mL with Milli-Q water and was poured in φ5-mm silicon tubes, followed by lyophilization with DC800 (Yamato Co., Ltd., Tokyo, Japan) to produce the EGCG-GSs or GS. To fabricate vhEGCG-GSs and vhGS, EGCG-GSs and GS were treated by vacuum heating using ETTAS AVO-250NS (AS ONE, Osaka, Japan) at 150 °C for 24 h with a gauge pressure of −0.1 MPa. All sponges were stored at 4 °C in the dark until use. 

### 4.2. Characterization of Sponges

SEM (FE-SEM, S-4800; Hitachi, Tokyo, Japan) was used to evaluate the morphological structure of the sponges. The existence of EGCG and the effect of vacuum heating were identified by absorbance spectra using attenuated total reflection Fourier-transform infrared spectroscopy (Perkin Elmer Spectrum One; PerkinElmer, Inc., Waltham, MA, USA). The sponges were analyzed over a range of 1800 to 500 cm^−1^ with 4 cm^−1^ resolution. 

### 4.3. Degradation Assay of Sponges

To verify the effect of vacuum heating, we performed degradation assays using sponges. Each sample weighing 2 mg was immersed in 300 µL of phosphate-buffered saline (PBS) and incubated in a shaking incubator (Taitec BR-40LF; Taitec Co., Ltd., Saitama, Japan) at 37 °C. A BCA Protein Assay Kit (Thermo Fisher Scientific, Inc., Waltham, MA, USA) was used to measure the concentration of the disintegrated gelatin in the supernatant using a SpectraMax M5 Multi-detection Microplate Reader System (Molecular Devices, LLC, San Jose, CA, USA) at 562 nm.

### 4.4. Animal Experiments

All animal experiments were approved by and strictly conformed to the guidelines of the Local Ethics Committee of Osaka Dental University (Approval No. 16-03013). Critical-sized defects (9 mm in diameter) of rat calvaria in Sprague–Dawley rats (male, 8 weeks old) were used to evaluate the bone-forming ability of each sample. Rats were anesthetized pre-operatively with pentobarbital and isoflurane inhalation. An L-shaped incision was made in the skin and periosteum to circumvent the defect area to avoid disruption of the bone-forming process. The critical-sized defects in the center of calvaria were prepared as reported previously using a trephine bur (Dentech, Tokyo, Japan) [47]. Seven columnar sponges (φ5 × 2 mm) for each sample condition (Table 1) were implanted into each defect; the periosteum and skin were overlaid and sutured firmly. Sample numbers 2 to 6 (Table 1) were utilized for the animal experiments. Defects without implantation were used as a negative control. At 4 and 8 wk after implantation, the treated calvariae were harvested to verify the bone-forming ability of each sponge. Five rats were used for each group. We used 35 rats in total for the experiments (4 wk: 5 rats × 6 groups including no implant; 8 wk: 5 rats for vhEGCG[0.07]-GS group). To analyze the morphology of newly formed bone in defects, the treated calvariae were examined by micro-computed tomography scanning (SMX-130CT; Shimadzu, Kyoto, Japan) at 76 μA of 57 kV radiation. Images were saved at a resolution of 512 × 512 pixels. TRI/3D-Bon (Ratoc System Engineering, Tokyo, Japan) was used to reconstruct the vertical and lateral views of the calvaria. BMD representing calcified bone tissue was assayed using cylindrical phantoms containing hydroxyapatite (hydroxyapatite content: 200–800 mg/cm^3^). BV/TV, BMC/TV, and BMD were calculated to assess the mineralized tissue volume, weight, and density in defects. For the histological evaluation, each calvaria was fixed with 4% paraformaldehyde phosphate buffer solution, decalcified, dehydrated, and embedded in paraffin. Thin sections (5 μm in thickness) were prepared and stained with hematoxylin–eosin. All images were captured using the BZ-9000 digital microscope (Keyence Co., Osaka, Japan).

### 4.5. Immunostaining and Scanning Electron Microscopy of Cells

The cell line UMR106 (CRL-1661), with osteoblast-like characteristics, was purchased from ATCC (Manassas, VA, USA). The cells were maintained at subconfluence in Dulbecco’s Modified Eagle Medium containing 10% fetal bovine serum and 1% antibiotics at 37 °C in 5% CO_2_. For SEM observation, 3 × 10^3^ cells were seeded onto the vhEGCG[0.07]-GS (φ5 × 10 mm) in a cryotube and rotated for 1 h to attach the cells. After 72 h of incubation, cells were washed twice with PBS, followed by fixation using 2% glutaraldehyde in 0.1 M PBS for 24 h at room temperature. The specimens were then sequentially dehydrated with various concentrations of ethyl alcohol and coated with OsO_4_ using the HPC-20 osmium Coater (Vacuum Device Co., Ltd., Ibaraki, Japan). FE-SEM (S-4800, Hitachi) was used to capture images. For immunostaining, 3 × 10^4^ cells were seeded onto the vhEGCG[0.07]-GS (φ5 × 10 mm) in a 24-well plate. At the indicated time points, the cells were fixed with 4% paraformaldehyde phosphate buffer solution. After washing with PBS, the fixed cells were permeabilized with 1% Triton X-100 in PBS. Actin was stained with Alexa Fluor 488 phalloidin (1:200; Thermo Fisher Scientific Inc., Waltham, MA, USA). Nuclear staining and mounting were performed using DAPI Fluoromount-G (SouthernBiotech, Birmingham, AL, USA).

### 4.6. Statistical Analysis

Statistical analyses were performed using BellCurve (Social Survey Research Information Co., Ltd., Tokyo, Japan). All results are presented as the mean ± standard deviation (SD). Statistical significance was calculated using a one-way analysis of variance, followed by a Tukey–Kramer test. Statistical significance was set at *p* < 0.01 and *p* < 0.05. 

## 5. Conclusions

We developed a novel bone substitute material, i.e., vacuum-heated EGCG-modified gelatin sponges, for bone regenerative therapy. Our results demonstrate that vacuum heating enhances the bone-forming ability of EGCG-GS. Despite the need to further optimize vacuum heating conditions, the results of in vitro experiments suggest that the observed increment in bone formation after vacuum heating can be partially attributed to the effect of attenuated degradability of the sponge via DHT cross-linking, which provides a scaffold for cells. Additionally, although there was no statistical difference among the effects of EGCG doses of 0.07 to 6.7 for bone formation, there was obvious increment of bone formation between EGCG doses of 0 and 0.07 (vhGS vs. vhEGCG[0.07]-GS). The results support that the pharmacological effect of EGCG has been retained even after the vacuum heating and is associated with the increment of bone formation. These findings indicate that vacuum heating can be a potential platform for the fabrication of other biomaterials containing EGCG for bone regeneration therapy. 

## Figures and Tables

**Table 1:** Synthesis conditions for sponges.

Sample No.	Sample Name	Gelatin (mg)	EGCG (mg)	Vacuum Heating
1	GS	100	0	-
2	vhGS	100	0	+
3	EGCG[0.07]-GS	100	0.07	-
4	vhEGCG[0.07]-GS	100	0.07	+
5	vhEGCG[0.7]-GS	100	0.7	+
6	vhEGCG[6.7]-GS	100	6.7	+

## Supplementary Materials

The following are available online, Figure S1: Characteristics of vhEGCG[0.7]-GS and vhEGCG[6.7]-GS, Figure S2: Robustness of sponges.
