# Supplementary material for: Epigallocatechin Gallate-Modified Gelatin Sponges Treated by Vacuum Heating as a Novel Scaffold for Bone Tissue Engineering
Source: Molecules. 2018 Apr 11;23(4):876. doi: 10.3390/molecules23040876 (PMC6017288; doi:10.3390/molecules23040876)

Supplementary Materials

Figure S1: Characteristics of vhEGCG[0.7]-GS and vhEGCG[6.7]-GS. A: Scanning electron microscope image of sponges. Bar: 100 µm. B: Absorbance spectra of sponges evaluated with attenuated total reflection Fourier-transform infrared spectroscopy. C: Degradation assay of sponges. Each sample weighing 2 mg was immersed in 300 µL of phosphate-buffered saline and incubated in a shaking incubator (Taitec BR-40LF; Taitec Co., Ltd., Saitama, Japan) for 48 h at 37°C. Mean and standard deviation (n = 3, ANOVA with Tukey-Kramer test). N.S.: not significant. D: Histological images of the defects treated with vhEGCG[0.7]-GS and vhEGCG[6.7]-GS, taken 4 wk after implantation. Bar: 600 µm.

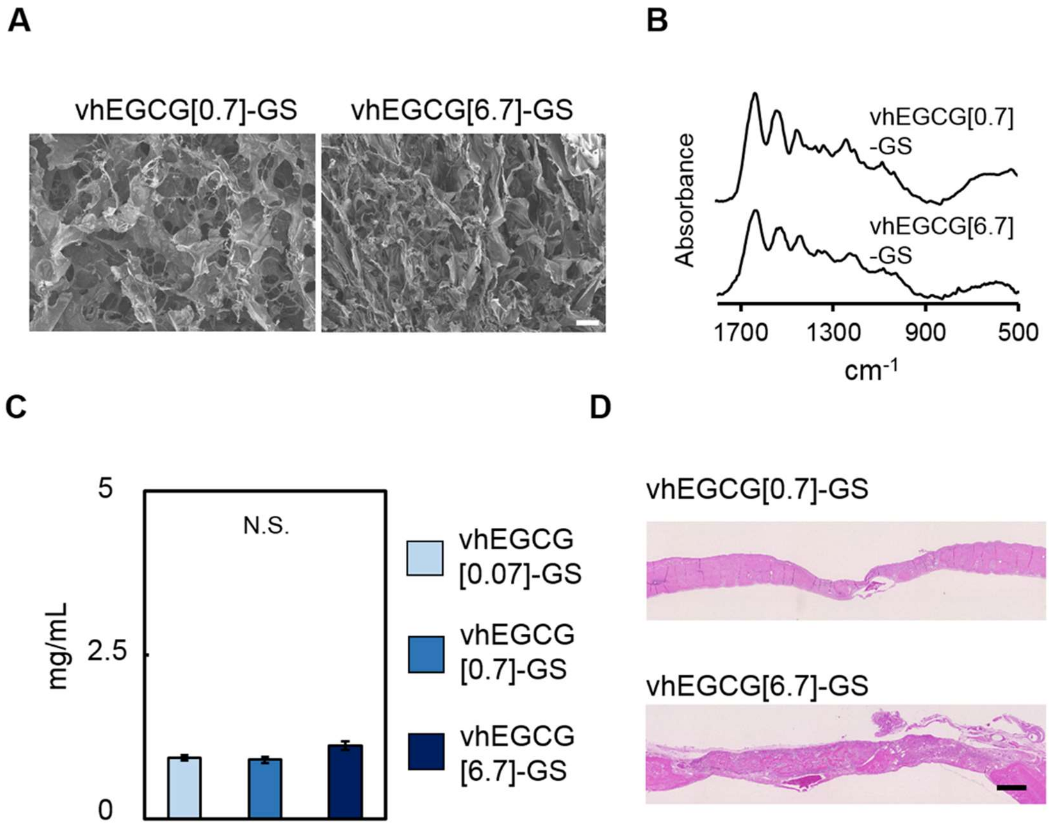

Figure S2: Robustness of sponges. Low- and high-magnification macroscopic images of EGCG[0.07]-GS and vhEGCG[0.07]-GS after 1 h of incubation in cell culture media. Arrows: margin of vhEGCG[0.07]-GS.

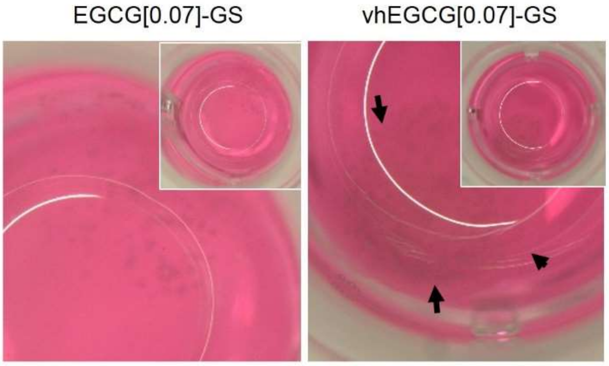

Supplement: Supplementary file 1 [file molecules-23-00876-s001.pdf]
